# Supplementary material for: Characterization of Purple Carrot Germplasm for Antioxidant Capacity and Root Concentration of Anthocyanins, Phenolics, and Carotenoids
Source: Plants (Basel). 2023 Apr 27;12(9):1796. doi: 10.3390/plants12091796 (PMC10181440; doi:10.3390/plants12091796)
Supplement: Supplementary file 1 [file plants-12-01796-s001.zip › plants-2334296-supplementary.pdf]

SUPPLEMENTARY MATERIAL

Characterization of Purple Carrot Germplasm for Antioxidant Capacity and Root Concentration of Anthocyanins, Phenolics, and Carotenoids

María Belén Pérez, Sofía Carvajal, Vanesa Beretta, Florencia Bannoud, María Florencia Fangio, Federico Berli, Ariel Fontana, María Victoria Salomón, Roxana Gonzalez, Lucia Valerga, Jorgelina C. Altamirano, Mehtap Yildiz, Massimo Iorizzo, Philipp W. Simon and Pablo F. Cavagnaro

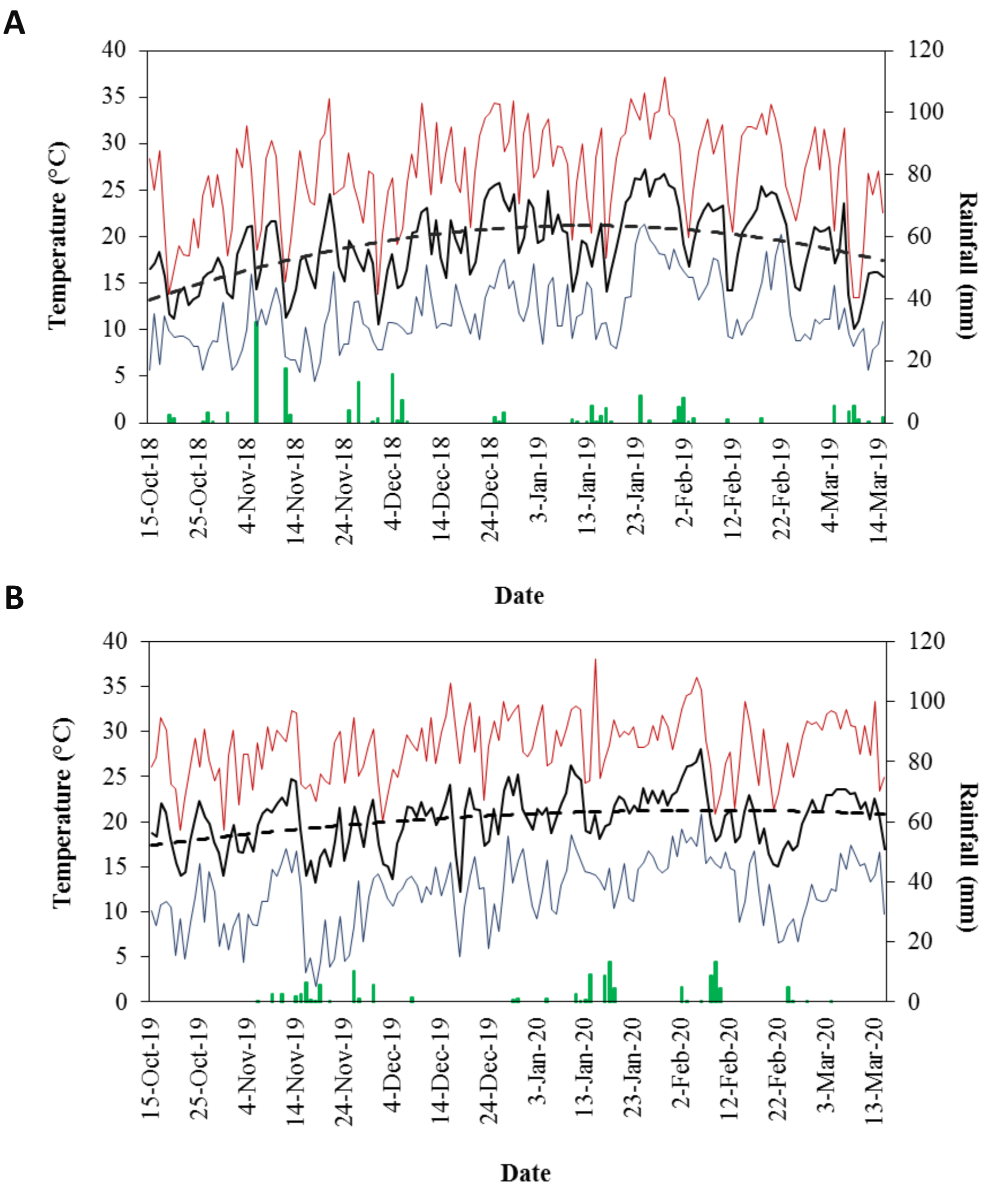

Supplementary Figure S1. Time-course variation for maximum (red line), minimum (blue line), and mean day temperature (black line) with adjusted trend line (black dashed line), and rainfall (green bars), at the cultivation site (Lujan de Cuyo, Mendoza, Argentina) for the period from carrot sowing to harvest (October 15 – March 15).

Supplementary Table S1. Variation for root concentration of individual and combined anthocyanin pigments for 27 carrot accessions, by year of cultivation.

**Acc.**[ζ]	**Year**	**Total ANT**[#] **Mean ± SE**	**Sum AA Mean ± SE (%)**	**Sum NAA Mean ± SE (%)**	**Cy3XG Mean (%)**	**Cy3XGG Mean (%)**	**Cy3XFGG Mean (%)**	**Cy3XSGG Mean (%)**	**Cy3XCGG Mean (%)**
	2018	3014.0 ± 236.3	2230.4 ± 250.6 (74.0)	783.6 ± 209.9 (26.0)	678.5 (22.5)	105.1 (3.5)	1746.4 (57.9)	287.1 (9.5)	196.8 (6.5)
**1**	2019	3330.1 ± 440.4	2647.4 ± 133.2 (79.5)	682.7 ± 343.0 (20.5)	601.8 (18.1)	80.9 (2.4)	1986.1 (59.6)	244.0 (7.3)	417.3 (12.5)
	Mean	3172.1 ± 338.4	2438.9 ± 191.9 (76.8)	733.2 ± 276.5 (23.2)	640.2 (20.2)	93.0 (2.9)	1866.3 (58.8)	265.6 (8.4)	307.1 (9.7)
	2018	2004.7 ± 169.7	1794.9 ± 165.5 (89.5)	209.8 ± 75.8 (10.5)	77.9 (3.9)	131.9 (6.6)	1398.3 (69.8)	281.2 (14.0)	115.4 (5.8)
**2**	2019	1443.4 ± 124.0	1188.0 ± 99.2 (82.3)	255.4 ± 291.1 (17.7)	183.2 (12.7)	72.2 (5.0)	1034.1 (71.6)	74.5 (5.2)	79.4 (5.5)
	Mean	1724.1 ± 146.9	1491.5 ± 132.4 (85.9)	232.6 ± 183.5 (14.1)	130.6 (7.6)	102.1 (5.9)	1216.2 (70.5)	177.9 (10.3)	97.4 (5.6)
	2018	1904.4 ± 311.9	1592.9 ± 248.6 (83.6)	311.4 ± 77.9 (16.4)	154.1 (8.1)	157.3 (8.3)	100.2 (5.3)	1483.8 (77.9)	8.9 (0.5)
**3**	2019	1831.3 ± 242.0	1489.7 ± 285.2 (81.3)	341.6 ± 77.0 (18.7)	168.1 (9.2)	173.5 (9.5)	305.9 (16.7)	1159.0 (63.3)	24.8 (1.4)
	Mean	1867.9 ± 277.0	1541.3 ± 266.9 (82.5)	326.5 ± 77.5 (17.5)	161.1 (8.6)	165.4 (8.9)	203.1 (10.9)	1321.4 (70.7)	16.9 (0.9)
	2018	1768.0 ± 126.5	1476.3 ± 110.9 (83.5)	291.7 ± 67.2 (16.5)	226.4 (12.8)	65.2 (3.7)	1139.4 (64.4)	252.2 (14.3)	84.8 (4.8)
**4**	2019	2027.0 ± 409.4	1270.0 ± 300.7 (61.5)	779.0 ± 114.4 (38.5)	588.6 (29.0)	190.4 (9.4)	1076.3 (53.1)	86.7 (4.3)	83.1 (4.1)
	Mean	1897.5 ± 268.0	1373.2 ± 205.8 (72.5)	535.4 ± 90.8 (27.5)	407.5 (21.5)	127.8 (6.7)	1107.9 (58.4)	169.5 (8.9)	84.0 (4.4)
	2018	1770.9 ± 273.3	1008.9 ± 189.1 (54.2)	752.0 ± 131.3 (45.8)	558.6 (31.5)	253.4 (14.3)	336.5 (19.0)	600.0 (33.9)	22.5 (1.3)
**5**	2019	2015.1 ± 268.7	1165.2 ± 170.8 (56.8)	849.9 ± 98.2 (43.2)	645.8 (32.0)	224.1 (11.1)	508.9 (25.3)	618.7 (30.7)	17.6 (0.9)
	Mean	1893.0 ± 271.0	1087.1 ± 180.0 (55.5)	801.0 ± 114.8 (44.5)	602.2 (31.8)	238.8 (12.6)	422.7 (22.3)	609.4 (32.2)	20.1 (1.1)
	2018	1464.3 ± 326.1	1170.0 ± 390.5 (76.5)	294.3 ± 67.1 (23.5)	230.4 (15.7)	113.9 (7.8)	153.1 (10.5)	962.5 (65.7)	4.4 (0.3)
**6**	2019	1603.6 ± 312.8	1348.8 ± 254.2 (84.7)	254.8 ± 78.2 (15.3)	173.1 (10.8)	81.8 (5.1)	131.7 (8.2)	1202.9 (75.0)	14.1 (0.9)
	Mean	1534.0 ± 319.5	1259.4 ± 322.4 (80.6)	274.6 ± 72.7 (19.4)	201.8 (13.2)	97.9 (6.4)	142.4 (9.3)	1082.7 (70.6)	9.3 (0.6)
	2018	1299.3 ± 136.7	1242.6 ± 124.5 (95.6)	56.7 ± 14.6 (4.4)	29.9 (2.3)	26.8 (2.1)	970.3 (74.7)	257.6 (19.8)	14.7 (1.1)
**7**	2019	1504.8 ± 213.0	1402.1 ± 207.1 (93.2)	102.7 ± 9.7 (6.8)	72.7 (4.8)	29.9 (2.0)	1221.5 (81.2)	88.0 (5.8)	92.6 (6.2)
	Mean	1402.1 ± 174.9	1322.4 ± 165.8 (94.4)	79.7 ± 12.2 (5.6)	51.3 (3.7)	28.4 (2.0)	1095.9 (78.2)	172.8 (12.3)	53.7 (3.8)
	2018	820.2 ± 94.8	798.4 ± 100.3 (97.5)	21.8 ± 8.4 (2.5)	17.5 (2.1)	4.4 (0.5)	194.8 (24.8)	594.6 (71.5)	9.0 (1.1)
**8**	2019	511.4 ± 124.3	496.5 ± 127.9 (96.6)	14.9 ± 5.2 (3.4)	9.7 (1.9)	5.2 (1.0)	121.3 (22.7)	369.5 (73.3)	5.7 (1.1)
	Mean	665.8 ± 109.6	647.5 ± 114.1 (97.1)	18.4 ± 6.8 (2.9)	13.6 (2.0)	4.8 (0.7)	158.1 (23.7)	482.1 (72.4)	7.4 (1.1)
**9**	2019	628.1 ± 179.7	590.0 ± 149.5 (94.0)	37.7 ± 30.2 (6.0)	28.9 (4.6)	8.8 (1.4)	513.6 (81.8)	76.7 (12.2)	nd (0.0)
**10**	2019	411.4 ± 101.8	355.0 ± 91.1 (86.3)	56.4 ± 10.7 (13.7)	56.4 (13.7)	0.0 (0.0)	354.0 (86.0)	0.0 (0.0)	1.0 (0.2)
	2018	345.4 ± 44.5	334.2 ± 42.9 (95.6)	9.1 ± 1.7 (4.4)	5.1 (1.5)	10.1 (2.9)	302.2 (87.5)	13.2 (3.8)	14.8 (4.3)
**11**	2019	480.3 ± 85.8	452.7 ± 82.6 (98.7)	12.5 ± 3.2 (1.3)	2.3 (0.5)	5.2 (1.1)	425.2 (88.5)	27.6 (5.7)	20.0 (4.2)
	Mean	412.9 ± 65.2	393.5 ± 62.8 (97.2)	10.8 ± 2.5 (2.8)	3.7 (0.9)	7.7 (1.9)	363.7 (88.1)	20.4 (4.9)	17.4 (4.2)
	2018	295.5 ± 32.4	268.8 ± 25.9 (90.9)	26.8 ± 2.6 (9.1)	15.9 (5.4)	10.8 (3.7)	212.7 (72.0)	54.0 (18.3)	2.0 (0.7)
**12**	2019	386.8 ± 52.0	336.7 ± 39.1 (87.0)	50.1 ± 15.1 (13.0)	29.2 (7.5)	20.9 (5.4)	286.2 (74.0)	33.1 (8.6)	17.4 (4.5)
	Mean	341.2 ± 42.2	302.8 ± 32.5 (89.0)	38.5 ± 8.9 (11.0)	22.6 (6.6)	15.9 (4.6)	249.5 (73.1)	43.6 (12.8)	9.7 (2.8)
	2018	185.7 ± 45.0	187.8 ± 44.0 (98.3)	2.9 ± 1.1 (1.7)	1.3 (0.7)	1.6 (0.9)	150.8 (81.2)	28.4 (15.3)	3.6 (1.9)
**13**	2019	375.8 ± 70.0	360.9 ± 69.5 (99.1)	4.8 ± 1.5 (0.9)	1.1 (0.3)	2.7 (0.7)	312.3 (83.1)	52.9 (14.1)	6.7 (1.8)
	Mean	280.8 ± 57.5	274.4 ± 56.8 (98.7)	3.9 ± 1.3 (1.3)	1.2 (0.4)	2.2 (0.8)	231.6 (82.5)	40.7 (14.5)	5.2 (1.8)
	2018	246.7 ± 55.0	217.8 ± 38.8 (88.2)	29.1 ± 12.3 (11.8)	27.1 (11.0)	1.8 (0.7)	149.7 (60.7)	61.1 (24.8)	7.0 (2.8)
**14**	2019	294.9 ± 57.5	266.2 ± 56.9 (91.4)	28.7 ± 8.6 (8.6)	23.0 (7.8)	2.3 (0.8)	175.5 (59.5)	87.9 (29.8)	6.1 (2.1)
	Mean	270.8 ± 56.3	242.0 ± 47.9 (89.8)	28.9 ± 10.5 (10.2)	25.1 (9.3)	2.1 (0.8)	162.6 (60.0)	74.5 (27.5)	6.6 (2.4)
**15**	2018	235.2 ± 34.8	221.2 ± 31.8 (94.0)	14.4 ± 4.2 (6.0)	8.9 (3.8)	5.1 (2.2)	203.0 (86.3)	15.4 (6.5)	2.8 (1.2)

Supplementary Table S1 - continued

Acc.[ζ]	Year	Total ANT[#] Mean ± SE	Sum AA Mean ± SE (%)	Sum NAA Mean ± SE (%)	Cy3XG Mean (%)	Cy3XGG Mean (%)	Cy3XFGG Mean (%)	Cy3XSGG Mean (%)	Cy3XCGG Mean (%)
	2018	95.6 ± 8.1	81.1 ± 5.0 (85.3)	14.5 ± 4.7 (14.7)	11.2 (11.7)	3.3 (3.5)	66.5 (69.6)	13.5 (14.1)	1.0 (1.0)
**16**	2019	84.2 ± 11.1	71.0 ± 8.9 (83.6)	13.2 ± 4.9 (16.4)	10.1 (12.0)	3.1 (3.7)	59.4 (70.5)	10.7 (12.7)	0.9 (1.1)
	Mean	89.9 ± 9.6	76.1 ± 7.0 (84.5)	13.9 ± 4.8 (15.5)	10.7 (11.8)	3.2 (3.6)	63.0 (70.0)	12.1 (13.5)	1.0 (1.1)
**17**	2019	80.4 ± 16.5	80.4 ± 16.5 (100)	0.0 ± 0.0 (0.0)	nd (0.0)	nd (0.0)	78.9 (98.1)	1.5 (1.9)	nd (0.0)
	2018	51.5 ± 10.7	47.9 ± 10.3 (92.7)	3.6 ± 0.6 (7.3)	2.5 (5.0)	1.1 (2.1)	33.6 (65.2)	13.4 (26.0)	0.9 (1.7)
**18**	2019	26.5 ± 4.6	25.0 ± 4.3 (94.4)	1.5 ± 0.3 (5.6)	0.9 (3.5)	0.6 (2.3)	16.8 (63.4)	7.7 (29.1)	0.5 (1.9)
	Mean	39.0 ± 7.7	36.5 ± 7.3 (93.6)	2.6 ± 0.5 (6.4)	1.7 (4.4)	0.9 (2.2)	25.2 (64.6)	10.6 (27.1)	0.7 (1.8)
	2018	40.5 ± 10.0	38.7 ± 9.9 (95.7)	1.7 ± 0.3 (4.3)	1.0 (2.5)	0.8 (2.0)	20.3 (50.1)	17.9 (44.2)	0.5 (1.2)
**19**	2019	34.1 ± 8.4	32.4 ± 8.1 (94.5)	1.7 ± 0.4 (5.5)	0.7 (2.1)	1.0 (2.9)	17.2 (50.4)	14.2 (41.6)	1.0 (2.9)
	Mean	37.3 ± 9.2	35.6 ± 9.0 (95.1)	1.7 ± 0.4 (4.9)	0.9 (2.3)	0.9 (2.4)	18.8 (50.3)	16.1 (43.0)	0.8 (2.0)
**20**	2019	29.6 ± 9.9	29.2 ± 9.5 (99.2)	0.4 ± 0.3 (0.8)	0.4 (1.4)	0.0 (0.0)	29.2 (98.6)	nd (0.0)	nd (0.0)
	2018	28.9 ± 9.2	25.5 ± 7.7 (88.2)	3.4 ± 1.6 (11.8)	0.6 (2.1)	2.8 (9.7)	14.5 (50.2)	10.8 (37.4)	0.2 (0.7)
**21**	2019	32.0 ± 10.9	27.1 ± 8.4 (84.1)	5.0 ± 2.5 (5.9)	3.2 (10.0)	1.9 (5.9)	17.5 (54.7)	8.7 (27.2)	0.7 (2.2)
	Mean	30.5 ± 10.1	26.3 ± 8.1 (86.2)	4.2 ± 2.1 (13.8)	1.9 (6.2)	2.4 (7.7)	16.0 (52.5)	9.8 (32.0)	0.5 (1.5)
	2018	27.0 ± 7.9	24.9 ± 7.7 (91.4)	2.1 ± 0.7 (8.6)	1.4 (5.2)	0.7 (2.6)	13.9 (51.5)	10.8 (40.0)	0.2 (0.7)
**22**	2019	19.0 ± 3.9	17.9 ± 4.2 (93.7)	1.1 ± 0.4 (6.3)	0.8 (4.2)	0.4 (2.1)	9.8 (51.6)	7.9 (41.6)	0.1 (0.5)
	Mean	23.0 ± 5.9	21.4 ± 6.0 (92.6)	1.6 ± 0.6 (7.4)	1.1 (4.8)	0.6 (2.4)	11.9 (51.5)	9.4 (40.7)	0.2 (0.7)
**23**	2019	25.8 ± 7.89	21.8 ± 6.1 (84.6)	4.0 ± 1.9 (15.4)	2.4 (9.3)	1.6 (6.1)	14.5 (56.2)	6.8 (26.4)	0.5 (2.1)
	2018	19.1 ± 5.90	18.3 ± 6.2 (94.6)	0.3 ± 0.2 (5.4)	0.4 (1.8)	0.3 (1.8)	7.6 (39.6)	10.5 (55.1)	0.3 (1.3)
**24**	2019	30.1 ± 6.63	29.2 ± 7.1 (97.2)	1.0 ± 0.5 (2.8)	0.6 (2.0)	0.3 (1.1)	12.4 (41.1)	16.4 (54.6)	0.4 (1.2)
	Mean	24.6 ± 6.27	23.7 ± 6.7 (95.9)	0.6 ± 0.3 (4.1)	0.5 (2.0)	0.3 (1.4)	10.0 (40.5)	13.5 (54.8)	0.3 (1.3)
**25**	2019	18.5 ± 5.6	18.5 ± 5.6 (100)	0.0 ± 0.0 (0.0)	nd (0.0)	nd (0.0)	nd (0.0)	18.5 (100)	nd (0.0)
**26**	2019	2.1 ± 0.4	2.1 ± 0.4 (100)	0.0 ± 0.0 (0.0)	nd (0.0)	nd (0.0)	2.1 (100)	nd (0.0)	nd (0.0)
	2018	1.5 ± 0.6	1.3 ± 0.5 (86.5)	0.2 ± 0.2 (13.5)	0.2 (10.8)	0.04 (2.7)	1.1 (74.3)	0.15 (10.1)	0.03 (2.0)
**27**	2019	0.8 ± 0.1	0.6 ± 0.1 (79.1)	0.2 ± 0.0 (20.9)	0.1 (16.9)	0.03 (3.9)	0.5 (68.8)	0.06 (7.8)	0.02 (2.6)
	Mean	1.1 ± 0.4	0.9 ± 0.3 (82.8)	0.2 ± 0.1 (17.2)	0.1 (12.9)	0.04 (3.1)	0.8 (72.4)	0.11 (9.3)	0.03 (2.2)

[ζ] Accession numbers refer to the carrot materials described in Table 1. Values are means of three replicates, expressed as mg kg^{-1} fw ± SE. Percentages (%) are relative to the total anthocyanin content. AA. acylated anthocyanins; NAA. non-acylated anthocyanins; nd. not detected; Cy3XG. cyanidin-3-(2″-xylose-galactoside); Cy3XGG. cyanidin-3-(2″-xylose-6-glucose-galactoside); Cy3XFGG. Cyanidin-3-(2″-xylose-6″-feruloyl-glucose-galactoside); Cy3XSGG. cyanidin-3-(2″-xylose-6″-sinapoyl-glucose-galactoside); Cy3XCGG. cyanidin-3-(2″-xylose-6″-(4-coumaroyl)glucose-galactoside). [#] Total anthocyanin content was calculated as the sum of the individual pigments quantified by HPLC analysis.

Supplementary Table S2. Edaphic characteristics of the carrot growing site

Edaphic parameter	Value (interpretation)
pH	7.5
Salinity (dS/m)	1.8 (not saline)
Total Nitrogen (N) (mg/kg)	1050 (high)
Organic matter (%)	5.6
Available phosphorus (P) (mg/kg)	2.7
Exchangeable potassium (K) (mg/kg)	205
C/N ratio	8.6
Texture	Silty loam
Sedimentation volume (cm^3/100g)	112

Soil analyses were performed at the Laboratory of Edaphology, Faculty of Agricultural Sciences, National University of Cuyo, Mendoza, Argentina.

Supplementary Table S3. Pairwise Pearson correlation coefficient values (r) among carrot bioactive compounds and antioxidant capacity for accessions 1-7, presenting high anthocyanin concentration (> 1000 mg kg^{-1} fw), for years 2018 and 2019

	**TAC$_{SPEC}$**	**TAC$_{HPLC}$**	**Cy3XG**	**Cy3XGG**	**Cy3XSGG**	**Cy3XFGG**	**Cy3XCGG**	**TAA**	**TNAA**	**TPC**	**ABTS**	**FRAP**	**DPPH**	**ORAC**	**β-carot**	**Lutein**
**TAC$_{SPEC}$**		0.80***	0.68***	0.29	0.04	0.52***	0.69***	0.63***	0.68***	0.87***	0.50**	0.50**	0.49**	0.27	0.13	0.01
**TAC$_{HPLC}$**	0.80***		0.67***	0.40	0.07	0.72***	0.86***	0.93***	0.69***	0.90***	0.57**	0.44**	0.50**	0.35*	0.34	0.04
**Cy3XG**	0.75***	0.64***		0.09	-0.19	0.41	0.62***	0.38*	0.98**	0.60**	0.41**	0.68***	0.37	0.24	0.15	-0.29
**Cy3XGG**	0.35**	0.44**	0.48*		0.48	-0.14	0.09	0.23*	0.39***	0.20	0.07	0.05	0.08	0.26	-0.07	-0.38
**Cy3XSGG**	0.33**	0.44**	-0.31	0.30		-0.56*	-0.38	0.35**	0.20	0.12	-0.16	-0.38	-0.41	-0.15	-0.51	-0.53
**Cy3XFGG**	0.41***	0.77***	0.57***	0.09	-0.59**		0.91***	0.75***	0.33*	0.63***	0.56*	0.41**	0.66***	0.36**	0.54**	0.67
**Cy3XCGG**	0.56***	0.86***	0.57***	0.08	-0.32**	0.91***		0.81***	0.57*	0.76***	0.54**	0.42**	0.64***	0.36**	0.61**	0.26
**TAA**	0.71***	0.80***	0.46	0.11	0.37**	0.76***	0.89***		0.38	0.86**	0.54***	0.26*	0.47*	0.31	0.22	0.38
**TNAA**	0.66***	0.80***	0.98***	0.64***	0.07	0.48*	0.48*	0.43*		0.58*	0.38*	0.62**	0.35*	0.27	0.24	0.11
**TPC**	0.77***	0.83***	0.56***	0.32*	0.01	0.65***	0.72***	0.80***	0.56**		0.47**	0.43***	0.43***	0.21	0.19	0.01
**ABTS**	0.48***	0.56***	0.42***	0.30*	0.24	0.57***	0.55***	0.50***	0.45**	0.52***		0.47**	0.75***	0.04	0.23	0.23
**FRAP**	0.38**	0.48***	0.44***	0.20	0.11	0.48***	0.45***	0.46***	0.36*	0.47***	0.44***		0.36**	0.44**	-0.20	-0.12
**DPPH**	-	-	-	-	-	-	-	-	-	-	-	-		0.02	0.30	0.41
**ORAC**	-	-	-	-	-	-	-	-	-	-	-	-	-		0.22	0.05
**β-carot.**	-	-	-	-	-	-	-	-	-	-	-	-	-	-		0.25
**Lutein**	-	-	-	-	-	-	-	-	-	-	-	-	-	-	-	

The diagonal gray boxes separate data for 2018 (upper half) and 2019 (lower half). TAC$_{SPEC}$: total anthocyanin content by spectrophotometry; TAC$_{HPLC}$: total anthocyanin content by HPLC; TAA: total acylated anthocyanins; TNAA: total non-acylated anthocyanins; TPC: total phenolics content. The full name of individual anthocyanin pigments (Cy3XG, Cy3XGG, Cy3XSGG, Cy3XFGG, Cy3XCGG) are presented in Table 2. Antioxidant capacity was estimated by four analytical methods (ABTS, FRAP, DPPH, and ORAC). *, **, *** indicate significant correlation at p<0.05, p<0.01, and p<0.001, respectively.

Supplementary Table S4. Pairwise Pearson correlation coefficient values (r) among carrot bioactive compounds and antioxidant capacity for accessions 8-17, presenting intermediate anthocyanin concentration (80-1000 mg kg^{-1} fw), for years 2018 and 2019

	TAC_{SPEC}	TAC_{HPLC}	Cy3XG	Cy3XGG	Cy3XSGG	Cy3XFGG	Cy3XCGG	TAA	TNAA	TPC	ABTS	FRAP	DPPH	ORAC	β-carot	Lutein
**TAC_{SPEC}**		0.76***	0.36**	0.42***	0.63***	0.49***	0.52***	0.57***	0.39***	0.78***	0.78***	0.43***	0.64***	0.38**	0.26	0.15
**TAC_{HPLC}**	0.60***		0.36**	0.46***	0.92***	0.41***	0.51***	0.98***	0.49***	0.82***	0.73***	0.54***	0.54***	0.51***	0.26	0.03
**Cy3XG**	0.56***	0.48***		0.36**	0.02	0.02	0.22	0.14	0.38**	0.39*	0.08	0.31*	0.19	0.05	0.24	0.26
**Cy3XGG**	0.48***	0.47***	0.39***		0.11	0.03	0.04	0.1	0.98***	0.65***	0.10	0.32*	0.26	0.15	0.12	0.07
**Cy3XSGG**	0.34***	0.44***	0.13	0.02		0.03	0.23	0.42***	0.10	0.05	0.83***	0.51***	0.68***	0.45**	0.01	0.10
**Cy3XFGG**	0.42***	0.83***	0.54***	0.45*	0.11		0.73**	0.92***	0.08	0.74***	0.43**	0.33**	0.37*	0.31**	0.12	0.16
**Cy3XCGG**	0.03	0.31*	0.03	0.48*	0.02	0.32**		0.50***	0.22	0.39***	0.40**	0.34**	0.36*	0.26*	0.12	0.29
**TAA**	0.56***	0.99***	0.39	0.42	0.49***	0.81***	0.32**		0.13	0.82***	0.73***	0.52***	0.64***	0.53***	0.25	0.13
**TNAA**	0.62***	0.55***	0.96***	0.64***	0.11	0.60*	0.16	0.46*		0.22	0.12	0.38*	0.29	0.14	0.21	0.28
**TPC**	0.45***	0.66***	0.35**	0.35**	0.34***	0.40**	0.15	0.65***	0.40**		0.76***	0.47***	0.64***	0.65***	0.03	0.08
**ABTS**	0.32*	0.44*	0.08	0.30*	0.01	0.81**	0.32**	0.35**	0.03	0.30*		0.56***	0.75***	0.49***	0.11	0.04
**FRAP**	0.35*	0.21*	0.04	0.06	0.47***	0.09	0.06	0.23*	0.05	0.27*	0.03		0.36**	0.48*	0.21	0.05
**DPPH**	-	-	-	-	-	-	-	-	-	-	-	-		0.11	0.39	0.03
**ORAC**	-	-	-	-	-	-	-	-	-	-	-	-	-		0.12	0.07
**β-carot.**	-	-	-	-	-	-	-	-	-	-	-	-	-	-		0.33
**Lutein**	-	-	-	-	-	-	-	-	-	-	-	-	-	-	-	

The diagonal gray boxes separate data for 2018 (upper half) and 2019 (lower half). TAC_{SPEC}: total anthocyanin content by spectrophotometry; TAC_{HPLC}: total anthocyanin content by HPLC; TAA: total acylated anthocyanins; TNAA: total non-acylated anthocyanins; TPC: total phenolics content. The full name of individual anthocyanin pigments (Cy3XG, Cy3XGG, Cy3XSGG, Cy3XFGG, Cy3XCGG) are presented in Table 2. Antioxidant capacity was estimated by four analytical methods (ABTS, FRAP, DPPH, and ORAC). *, **, *** indicate significant correlation at $p<0.05$, $p<0.01$, and $p<0.001$, respectively.

Supplementary Table S5. Pairwise Pearson correlation coefficient values (r) among carrot bioactive compounds and antioxidant capacity for accessions 18-29, presenting low anthocyanin concentration (< 80 mg kg^{-1} fw), for years 2018 and 2019

	TAC_{SPEC}	TAC_{HPLC}	Cy3XG	Cy3XGG	Cy3XSGG	Cy3XFGG	Cy3XCGG	TAA	TNAA	TPC	ABTS	FRAP	DPPH	ORAC	β-carot	Lutein
**TAC_{SPEC}**		0.37**	0.59***	0.39**	0.07	0.47***	0.46***	0.35**	0.53**	0.61***	0.09	0.21*	0.17*	0.23*	0.36	0.34
**TAC_{HPLC}**	0.53***		0.66***	0.46***	0.87***	0.96***	0.85***	0.99***	0.70***	0.66***	0.33**	0.37**	0.37**	0.23*	0.31	0.06
**Cy3XG**	0.22*	0.36**		0.17	0.33**	0.43*	0.72**	0.34*	0.85***	0.12	0.24	0.09	0.29*	0.08	0.25	0.30
**Cy3XGG**	0.06	0.55**	0.32**		0.17	0.43*	0.65***	0.42*	0.66***	0.10	0.41**	0.08	0.30*	0.42*	0.01	0.05
**Cy3XSGG**	0.02	0.53**	0.01	0.12		0.71**	0.65***	0.88***	0.42*	0.43***	0.27*	0.68**	0.57**	0.03	0.34	0.15
**Cy3XFGG**	0.32**	0.84***	0.52*	0.26	0.003		0.89***	0.96***	0.22	0.44***	0.18	0.49**	0.37**	0.09	0.35	0.42
**Cy3XCGG**	0.09	0.70***	0.67*	0.38*	0.34*	0.99***		0.86***	0.23	0.47***	0.43**	0.42*	0.27*	0.17	0.38	0.20
**TAA**	0.37*	0.99***	0.29*	0.29*	0.57**	0.65***	0.65**		0.20	0.47***	0.33**	0.36**	0.48**	0.05	0.37	0.34
**TNAA**	0.33*	0.47**	0.94***	0.63***	0.05	0.48*	0.68*	0.15		0.15	0.03	0.43*	0.43*	0.29*	0.29	0.13
**TPC**	0.23*	0.32**	0.08	0.08	0.49**	0.002	0.003	0.32**	0.50*		0.21*	0.01	0.01	0.27*	0.14	0.11
**ABTS**	0.26*	0.33**	0.07	0.07	0.30*	0.05	0.05	0.34**	0.09	0.35*		0.01	0.27*	0.51**	0.01	0.03
**FRAP**	0.08	0.23**	0.06	0.02	0.37*	0.11	0.11	0.26*	0.06	0.09	0.19		0.23*	0.11	0.28	0.30
**DPPH**	-	-	-	-	-	-	-	-	-	-	-	-		0.11	0.24	0.14
**ORAC**	-	-	-	-	-	-	-	-	-	-	-	-	-		0.04	0.09
**β-carot.**	-	-	-	-	-	-	-	-	-	-	-	-	-	-		0.50*
**Lutein**	-	-	-	-	-	-	-	-	-	-	-	-	-	-	-	

The diagonal gray boxes separate data for 2018 (upper half) and 2019 (lower half). TAC_{SPEC}: total anthocyanin content by spectrophotometry; TAC_{HPLC}: total anthocyanin content by HPLC; TAA: total acylated anthocyanins; TNAA: total non-acylated anthocyanins; TPC: total phenolics content. The full name of individual anthocyanin pigments (Cy3XG, Cy3XGG, Cy3XSGG, Cy3XFGG, Cy3XCGG) are presented in Table 2. Antioxidant capacity was estimated by four analytical methods (ABTS, FRAP, DPPH, and ORAC). *, **, *** indicate significant correlation at $p<0.05$, $p<0.01$, and $p<0.001$, respectively.

Supplementary Table S6. Cohen's d effect size estimates among sub-classes of carrot accessions with high (H) (>1000 mg kg^{-1} fw), intermediate (I) (80-1000 mg kg^{-1} fw), and low anthocyanin content (L) (<80 mg kg^{-1} fw), for 16 variables and two years.

		2018				2019		
		H	I	L		H	I	L
Cy3XGG	H	0.00	-	-	H	0.00	-	-
	I	1.95	0.00	-	M	1.99	0.00	-
	L	1.94	1.57	0.00	L	2.15	0.91	0.00
Cy3XCGG	H	0.00	-	-	H	0.00	-	-
	I	1.08	0.00	-	M	1.02	0.00	-
	L	1.13	1.36	0.00	L	1.11	1.09	0.00
Cy3XFGG	H	0.00	-	-	H	0.00	-	-
	I	1.37	0.00	-	M	1.37	0.00	-
	L	1.65	2.89	0.00	L	1.97	1.97	0.00
Cy3XG	H	0.00	-	-	H	0.00	-	-
	I	1.26	0.00	-	M	1.50	0.00	-
	L	1.26	1.11	0.00	L	1.63	1.15	0.00
Cy3XSGG	H	0.00	-	-	H	0.00	-	-
	I	1.16	0.00	-	M	1.14	0.00	-
	L	1.45	0.68	0.00	L	1.40	0.78	0.00
TAC_{SPEC}	H	0.00	-	-	H	0.00	-	-
	I	2.79	0.00	-	M	3.06	0.00	-
	L	3.45	1.82	0.00	L	3.83	2.24	0.00
TAC_{HPLC}	H	0.00	-	-	H	0.00	-	-
	I	2.54	0.00	-	M	2.51	0.00	-
	L	2.98	1.79	0.00	L	3.19	2.22	0.00
TAA	H	0.00	-	-	H	0.00	-	-
	I	2.44	0.00	-	M	2.36	0.00	-
	L	3.03	1.72	0.00	L	3.19	2.20	0.00
TNAA	H	0.00	-	-	H	0.00	-	-
	I	1.58	0.00	-	M	1.75	0.00	-
	L	1.58	1.43	0.00	L	1.90	1.24	0.00
TPC	H	0.00	-	-	H	0.00	-	-
	I	2.36	0.00	-	M	2.78	0.00	-
	L	3.11	1.36	0.00	L	4.45	1.92	0.00
ABTS	H	0.00	-	-	H	0.00	-	-
	I	2.13	0.00	-	M	2.02	0.00	-
	L	3.25	2.28	0.00	L	3.25	2.35	0.00
FRAP	H	0.00	-	-	H	0.00	-	-
	I	2.59	0.00	-	M	3.25	0.00	-
	L	3.61	1.91	0.00	L	3.95	1.30	0.00
DPPH	H	0.00	-	-				
	I	2.29	0.00	-				
	L	2.40	0.40	0.00				
ORAC	H	0.00						
	I	3.25	0.00					
	L	5.27	1.74	0.00				
Lutein	H	0.00	-	-				
	I	0.06	0.00	-				
	L	0.22	0.27	0.00				
β-carotene	H	0.00	-	-				
	I	2.20	0.00	-				
	L	1.22	0.26	0.00				
